# Biomarkers to guide sepsis management

**DOI:** 10.1186/s13613-025-01524-1

**Published:** 2025-07-21

**Authors:** Vasiliki Bourika, Evangelia-Areti Rekoumi, Evangelos J. Giamarellos-Bourboulis

**Affiliations:** 1 Hellenic Institute for the Study of Sepsis, Athens, Greece; 2 4th Department of Internal Medicine, National and Kapodistrian University of Athens, Athens, Greece

**Keywords:** Sepsis, Biomarkers, Guidance, Antibiotics, Fluids, Vasopressors

## Abstract

**Background:**

Sepsis remains a major cause of morbidity and mortality. Precision therapeutics are now regarded as a novel prospective to improve outcome. This approach relies on biomarkers to identify a pathway of pathogenesis which prevails and directs the best available therapeutic option to modulate this pathway. This review provides the most recent findings on biomarkers for bacterial or viral sepsis. These biomarkers provide guidance for prompt diagnosis and management tailored to specific needs.

**Main body:**

Keywords relative to sepsis management (early recognition, antibiotic administration, selection of fluids, vasopressors and immunotherapy) were searched across PubMed database. Published evidence the last five years exists for heparin-binding protein (HBP), monocyte distribution width (MDW), interleukin-10 (IL-10), presepsin, procalcitonin and C-reactive protein (CRP) for early sepsis diagnosis; procalcitonin is the most well-studied biomarker for antibiotic guidance. Endothelial and cardiac biomarkers have been explored as tools to tailor circulatory support in sepsis, including fluid therapy, and the targeted use of vasopressors for vascular tone optimization.

**Conclusion:**

This review explored how biomarkers can optimize immunomodulatory therapies, guide vasopressor initiation, inform antibiotic stewardship, and aid in fluid resuscitation decisions, ultimately improving patient outcomes.

**Supplementary Information:**

The online version contains supplementary material available at 10.1186/s13613-025-01524-1.

## Background

Despite scientific advances and research, sepsis remains one major worldwide health burden and a major cause of morbidity and mortality, affecting 48 million individuals annually and causing almost 12 million deaths [1]. Hospitalizations due to sepsis are rising, particularly among elderly and those with chronic comorbidities, leading to increased healthcare costs and resource utilization [2]. Even with improvements in critical care, sepsis is challenging to diagnose and treat quickly due to its diverse nature and heterogeneous clinical presentation [3].

Besides bacteria, infections with fungi, viruses, or parasites can also cause sepsis [4]. Severe parasitic infections, such as malaria and leishmaniasis, remain a significant cause of sepsis globally, especially in developing countries [5, 6]. Severe malaria has shown to meet clinical sepsis criteria, highlighting its role as a parasitic cause of sepsis in endemic settings [7]. However, due to limited availability of biomarker studies in parasitic sepsis, these infections were not included in the current manuscript.

Although this paper focuses on bacterial and viral sepsis, it is important to recognize that the host response differs significantly depending on the infection. As such, biomarker profiles may vary across sepsis subtypes. For example, in severe COVID-19 pneumonia or ARDS, blood lactate is typically within the normal range despite severe hypoxemia - which may imply maintained mitochondrial function - while HDL-c levels are significantly low [8, 9].

Despite limited data available among reports comparing viral to bacterial sepsis, some biomarkers like procalcitonin (PCT) and interferon-related proteins perform well to discriminate between bacterial and viral infections [10]. PCT has demonstrated diagnostic value in the detection of bacterial sepsis, with higher levels typically observed in Gram-negative compared to Gram-positive infections [11]. Additionally, the interferon-inducible Myxovirus resistance protein A (MxA) increases significantly during viral infections but remains low in bacterial ones. Its combination with CRP has demonstrated potential to distinguish viral from bacterial infections, further enhancing early sepsis stratification [12].

**Fig. 1:**
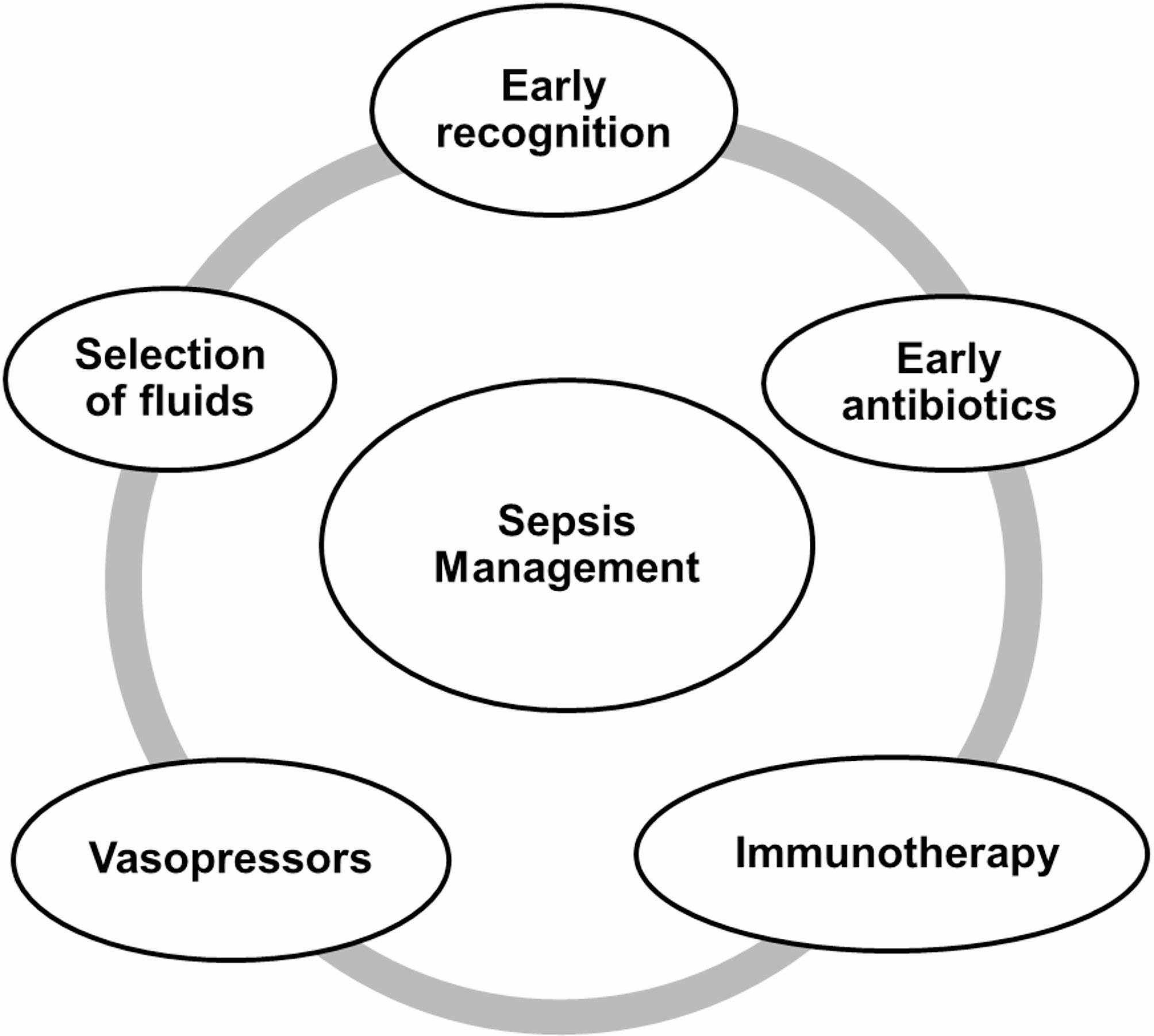
Principles of sepsis management

The principle of precision therapeutics is the use of biomarkers which may indicate a pathway of pathogenesis which prevails and directs the best available therapeutic option [13]. The biomarker is often a measurable variable which may indicate prognosis and suggest response to therapy [14]. Sepsis biomarkers are measures of metabolic stress, endothelial dysfunction, immunological dysregulation, and inflammation [15]. The purpose of this review is to compile the latest research results regarding biomarkers of bacterial or viral sepsis which facilitate prompt diagnosis and guide management tailored to specific needs (Fig. 1).

## Literature retrieval

We performed a structured literature search to identify biomarkers that inform different aspects of sepsis management. Keywords relative to sepsis management (early recognition, antibiotic administration, selection of fluids, vasopressors and immunotherapy) were searched across PubMed using MeSH terms for studies in humans, published between 1 January 2020 and 14 November 2024 in English - language. All terms were searched separately for bacterial and viral sepsis.

For early sepsis recognition, given the extensive number of biomarkers and related publications, we selected these based on their predictive value - specifically, their ability to anticipate the onset or clinical worsening of sepsis, rather than merely diagnose it after it has already developed. Studies addressing only early mortality or general prognosis were excluded. Also, given the time - sensitive nature of early recognition we prioritized biomarkers that are readily measurable in standard laboratory settings or already integrated into routine workflows. Additionally, biomarkers with strong pathophysiological relevance and translational potential, were included when supported by recent high-quality studies.

Although the primary focus was on studies published between 2020 and 2024, eight publications outside this range were included for their intellectual contribution and clinical impact. These consisted of seven landmark trials published before 2020 [36, 38, 59, 61]^68^– [70] and one 2025 meta-analysis on biomarker-guided corticosteroid therapy [58]. All records were screened and selected through a targeted screening process based on predefined criteria – as it presented in the supplementary table – and after title/abstract screening and full-text evaluation.

**Fig. 2:**
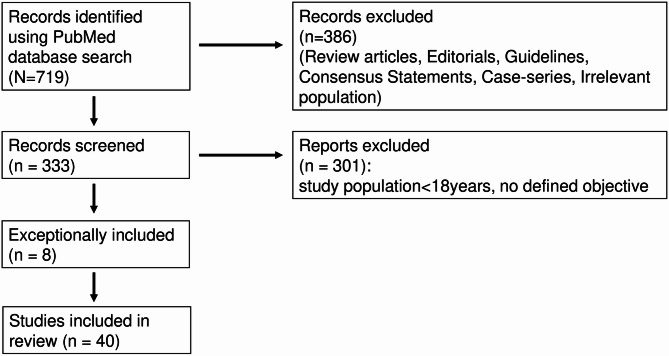
Retrieval process of literature research using the PubMed database for the review of biomarkers to guide sepsis management

The retrieval process is presented in the supplementary file. The detailed search process is presented in Fig. 2 and in the Supplement.

## Biomarkers for early recognition of sepsis

**Fig. 3:**
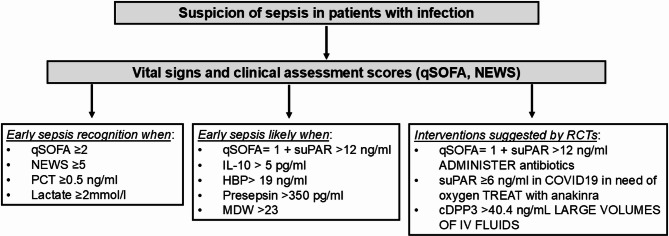
Suggested algorithm for early sepsis recognition and management

This manuscript focuses on the role of biomarkers in guiding key aspects of sepsis management. As stated by the Surviving Sepsis Campaign, the importance of promptly identifying and managing sepsis remains a core principle [16]. In this context, among the broad range of proposed biomarkers for early sepsis recognition, we focused on those with evidence supporting their ability to predict the imminent progression to sepsis in patients with suspected infection, thereby enabling timely intervention. From this perspective - balancing clinical applicability and biological relevance - published evidence over the last five years supports the use of heparin-binding protein (HBP), monocyte distribution width (MDW), interleukin-10 (IL-10), presepsin, procalcitonin (PCT) and, C-reactive protein (CRP). Figure 3.

### Heparin-binding protein

Bacterial products and inflammatory mediators lead to neutrophilic activation and release of stored heparin-binding protein (HBP). The antimicrobial, chemotactic and vascular permeability-enhancing capabilities of HBP are key contributors of organ dysfunction [17]. The PROMPT prospective, non-interventional, multi-center clinical study, aimed to evaluate the utility of HBP as diagnostic tool of sepsis in the emergency department (ED). The study population consisted of 371 adult patients with suspected infection and at least one of fever (> 38 °C) or hypothermia (< 36 °C), tachycardia (> 90 beats/min), tachypnea (> 20 breaths/min) and reported symptoms of fever and chills. Sepsis was defined by the Sepsis-3 criteria and patients who progressed into sepsis the first 72 h had significantly higher HBP in the ED compared to non-septic patients (*p* = 0.008). A threshold of 19.8 ng/ml had 66.3% sensitivity, 44.9% specificity, 49.3% positive predictive value (PPV) and 62.2% negative predictive value (NPV) for early sepsis diagnosis. Although the number of deaths was limited, the authors suggested that the same threshold resulted in 100% sensitivity and specificity for the prediction of death [18].

### Interleukin-10

A recent retrospective observational cohort study by Zhou et al. evaluated the combination of various biomarkers with clinical scores for the prediction of sepsis in 491 patients with confirmed infection and no signs of sepsis upon ED admission. 177 patients eventually developed sepsis and IL-10 levels were higher compared to patients with infection without sepsis (*p* < 0.001). The combination of IL-10 and the National Early Warning Score (NEWS) – a standardized scoring system based on standard vital signs measurements to identify early clinical deterioration – provided area under the curve 0.789 for early sepsis recognition translating into IL-10 ≥5.03 pg/mL and NEWS≥5 as the best screening tools [19, 20].

### Monocyte distribution width

Monocyte distribution width (MDW) is a blood test parameter included in the standard complete blood count (CBC) test [21]. Since monocytes enlarge in size upon activation in the event of bacteremia or fungemia, MDW has been suggested as a diagnostic tool of sepsis [22]. In a single-centre prospective cohort study, 402 patients admitted in the ED were allocated to four groups: 64 patients compromised the “non-infection” group, 82 patients the “infection” group, 202 the “infection + SIRS” group and 54 patients the “sepsis-3” group. For the prediction of sepsis, the authors suggested that MDW > 23.4 had sensitivity, 69.8% specificity 67.5%, positive predictive value (PPV) 25.5% and negative predictive value (NPV) 93.3% [16].

Another observational, prospective study by Polilli et al. investigated the role of MDW for sepsis prediction in 129 ICU patients. MDW was significantly higher in the septic group compared to patients without sepsis (median 25.6 vs. 21, *p* < 0.001) and MDW > 23 had 75.3% sensitivity, 88.7% specificity, 90.2% PPV and 72.3% NPV for sepsis diagnosis [22].

### Presepsin and procalcitonin

Presepsin is the soluble counterpart of CD14 the receptor for lipopolysaccharides (LPS). CD14 is expressed on multiple cell types implicated in the pathophysiology of sepsis and facilitates the transmission of LPS-mediated signals into the cells [23]. An observational single-centre cohort study in the ED in China was conducted investigating the role of various biomarkers in 198 patients with sepsis and 40 healthy controls. 77 out of 198 patients were diagnosed with septic shock. Presepsin and PCT levels were significantly elevated in patients with septic shock compared to those with sepsis (*p* < 0.001), and in non-survivors compared to survivors (*p* < 0.05). For presepsin, the AUC for the 28-day mortality prediction was 0.699 (0.619–0.780) (*p* < 0.001) and for procalcitonin 0.599 (0.515–0.683) (*p* = 0.021). The prognostic accuracy of both presepsin and procalcitonin improves with the addition of human leucocyte antigen (HLA-DR) [0.727 (0.651–0.803), *p* < 0.001 and 0.682 (0.603–0.761), *p* < 0.001, respectively]. The authors proposed that the combination of presepsin with HLA-DR may enhance prognostic utility during sepsis [24].

Procalcitonin has been extensively studied for its role in the early recognition of infection and sepsis [24]. PCT was used to improve sepsis diagnosis in patients scoring negative by the quick sequential organ failure assessment (qSOFA) score, in a multicenter, prospective, observational study. Concentrations higher than 0.25 µg/L had sensitivity 75.3%, specificity 80%, PPV 60.7%, NPV 88.7% and higher than 0.5 µg/L sensitivity 64.2%, specificity 89.6%, PPV 71.8%, NPV 85.9% for sepsis diagnosis. The pairing of procalcitonin and proadrenomedullin (proADM) improved the diagnostic accuracy for septic shock (AUC:0.86, 95% CI:0.67–0.93) [25]. Among other inflammatory markers, Yang et al. assessed retrospectively procalcitonin levels of 505 adults with bloodstream infection (BSI) and 102 with localized bacterial infection (LBI) and observed that PCT was significantly higher in BSI cases. With an AUC of 0.8835, a sensitivity of 73.1%, and a specificity of 87.2%, it emerged as a reliable tool for early BSI detection. The diagnostic potential for pathogen classification was further supported by animal model data, which was not included in this review due to pre-defined exclusion criteria [26]. 

### C-reactive protein

Recent studies evaluate C-reactive protein (CRP) in the ED for early diagnosis and prediction of sepsis progression. Christensen et al. assessed prospectively the contribution of various biomarkers in the early recognition of sepsis in the emergency department and CRP ≥ 50 mg/L provided PPV 95%; concentrations below 4 mg/L could reliably rule out infections (negative predictive value, NPV: 93%). No significant differences were observed between patients with bacterial and viral infections [27]. Patients with diffuse secondary peritonitis underwent blood sampling preoperatively and daily postoperatively; 100 patients who met the SIRS (systemic inflammatory response syndrome) criteria, were allocated to either the SIRS (45 patients) or sepsis (55 patients) groups. CRP preoperative values were significantly higher in the sepsis group (AUC:0.71, 95%CI: 0.61–0.81) [28].

The prognostic value of CRP in the ICU was examined retrospectively, by Koozi H et al. CRP values, from 6 h prior to 1 h after ICU admission, were followed-up in 819 patients. CRP was significantly higher among non-survivors with sepsis both with microbiology and non-microbiology proof of infection. Furthermore, patients who required ICU hospitalization for 3 days or more, had higher CRP levels compared to patients who were discharged early. The authors suggested that CRP more than 100 mg/L upon admission to the ICU is an independent negative predictor for both ICU and 30-day mortality, as well as length of stay, in patients who meet the Sepsis-3 definitions [29] (Table 1). In all studies, result interpretation is subject to confounding; potential confounders are listed in Table 2.

**Table 1:** Summary of original studies for biomarkers of early sepsis recognition

Reference	Biomarker(s)	Measurement	SIRS, Sepsis and Septic shock definition	Study population	Results
[16]	MDW Presepsin	Beckman Coulter DxH 900	Sepsis-3 criteria	402 patients in the ED: -“non-infection”: 64 -“infection”: 82 -“infection + SIRS”: 202 -“sepsis-3”: 54	**Prediction of infection + SIRS**: MDW > 20: AUC 0.753 (0.701–0.804). Sensitivity 86.4%, specificity 54.2%, PPV 76.4%, NPV 70% **Prediction of sepsis**: MDW > 23.4: AUC 0.722 (0.652–0.792) Sensitivity 69.8%, specificity 67.5%, PPV 25.5%, NPV 93.3%
[16, 18]	Heparin-binding protein (HBP)	Plasma, fluorescence dry quantitive immunoassay, Jet-iStar 800 analyzer (Join- Star, Hangzhou, China)	Sepsis-3 criteria	371 patients with suspected infection in the ED -166 developed sepsis within 72 h postadmission -205 non-sepsis	Higher HBP levels in patients who developed sepsis (*p* = 0.008) Diagnosis of sepsis: HBP > 19.8 ng/ml Sensitivity 66.3%, specificity 44.9%, PPV 49.3%, NPV 62.2%
[20]	IL-10	N/A	Sepsis-3 criteria	491 patients with confirmed infection and no evidence of sepsis upon ED admission: -177 sepsis group -314 infection group	Prediction of sepsis: IL-10 OR 2.20 (95% CI 1.78–2.71) NEWS OR 1.92 (95% CI 1.57–2.34) AUC (IL-10 + NEWS): 0.789 (*p* < 0.0001
[22]	MDW	UniCel DxH800 hematologic analyzer system (Beckman Coulter, Inc., Brea, California)	Sepsis-3 criteria	129 ICU patients: -74: sepsis group -55: no sepsis	Sepsis group vs. no sepsis: median MDW 25.6 vs. 21, *p* < 0.001 Prediction of sepsis: MDW AUC 0.84 (0.77–0.91) MDW > 23: Sensitivity 75.3%, specificity 88.7%, PPV 90.2%, NPV 72.3%
[24]	Presepsin Procalcitonin HLA-DR	Presepsin: PATHFAST automated immunoanalyser (Mitsubishi Chemical Medience Corp., Tokyo) PCT: BioMerieux Mini VIDAS immunoassay analyser (Block Scientific, Bohemia, NY) HLA-DR (expressed in MFI): flow cytometry, Gallios Flow Cytometer (Beckman Coulter, Brea, CA)	Sepsis-3 criteria	40 healthy controls 198 ED patients with sepsis 77/198 septic shock	Patients with sepsis vs. patients with septic shock: higher levels of presepsin and PCT (*p* < 0.001) Survivors vs. non-survivors: higher levels of presepsin and PCT (*p* < 0.05) AUC for 28-day mortality prediction: Presepsin: 0.699 (0.619–0.780), *p* < 0.001 Presepsin + HLA-DR MFI: 0.727 (0.651–0.803), *p* < 0.001 PCT: 0.599 (0.515–0.683), *p* = 0.021 PCT + HLA-DR MFI: 0.682 (0.603–0.761), *p* < 0.001
[25]	Procalcitonin, proADM	Automated immunofluorescent assays Procalcitonin: sensitive KRYPTOR proADM: KRYPTOR (BRAHMS GmbH, Hennigsdorf, Germany)	Sepsis-3 criteria	1426 ED patients with qSOFA ≥ 1: 1009 no septic patients 417 patients with sepsis	PCT for the prediction of sepsis: AUC:0.86, 95% CI:0.79–0.93 Cut-off values: 0.25 µg/L: sensitivity 75.3%, specificity 80%, PPV 60.7%, NPV 88.7% 0.5 µg/L sensitivity 64.2%, specificity 89.6%, PPV 71.8%, NPV 85.9% PCT and proADM for the prediction of septic shock: AUC:0.86, 95% CI:0.67–0.93
[26]	Procalcitonin IL-6 IL-10	Blood culture: Bact/ALERT 3D-120, BioMerieux, Marcy l’Etoile, France Procalcitonin: cobase e-602, Roche, Switzerland IL-6 and IL-10: DxFLEX, Beckman Coulter, USA	Sepsis-3 criteria	505 BSI patients analyzed (331 Gram-negative, 174 Gram positive); anaerobic/fungal cases excluded 102 patients with LBI	With a threshold of > 0.675 ng/ml, PCT distinguished BSI from LBI with an AUC of 0.8835, sensitivity of 73.1% and specificity of 87.2%.
[27]	CRP	Roche Cobas 8000 c702	Patients with possible sepsis	Admissions to ED were catecorized as: “not likely” group: 154 patients with no signs of infection “definite” group: 102 patients with a microbiologically confirmed infection “probable” group: 135 patients with signs of infection, not microbiologically confirmed	Diagnosis of infection in ED patients with possible sepsis: CRP AUC of 0.913 (95% CI: 0.884–0.942) CRP ≥ 50 mg/L PPV 95% or < 4 mg/L NPV 93%
[28]	CRP	Roche Cobas 6000 auto- mated analyzer (Roche Diagnostics, Mannheim, Germany)	Sepsis-2 criteria	100 patients: SIRS group 55 patients: SEPSA group	Preoperative CRP for differentiation SIRS/SEPSA: AUC:0.71, 95%CI: 0.61–0.81 CRP > 28.1 sensitivity 80%, specificity 60%
[29]	CRP	N/A	Sepsis-3 criteria	819 patients with sepsis 454/819: culture positive subgroup	↑ CRP levels in non-survivors compared to survivors: Sepsis group *p* = 0.023 Culture-positive subgroup *p* = 0.023 CRP > 100 mg/L: independent negative predictor

Abbreviations: AUC: area under the curve; BSI: Blood stream infection; CI: confidence interval; CRP: C-reactive protein; ED: emergency department; HLA-DR: human leucocyte antigen, IL-6: interleukin-6; IL-10: interleukin-10; LBI: Localized bacterial infection; MDW: monocyte distribution width; MFI: mean fluorescence intensity; N/A: not applicable; NEWS: National Early Warning Score; NPV: negative predictive value; PCT: procalcitonin; PPV: positive predictive value; proADM: pro- adrenomedullin; SIRS: systemic inflammatory response syndrome

**Table 2:** Factors that May influence interpretation of biomarkers

Reference	Biomarker	Time of blood sample	Prior corticosteroid treatment	Comorbidities
[16]	Monocyte distribution width	Not applicable (in the ED)	Not applicable	No difference in the underlying condition
[18]	Heparin-binding protein	Within 1 h of ED admission	No difference in corticosteroid treatment among septic and non-septic patients	There was difference among septic and non-septic patients for: -Type 2 diabetes -Chronic heart failure -Coronary heart disease -Chronis obstructive pulmonary disease -Chronic renal failure -Chemotherapy -Non metastatic solid tumor -Ischemic stroke -Atrial fibrillation -Dementia -Nephrolithiasis -Gallstones
[20]	Interleukin-10	Not applicable (in the ED)	Not applicable	There was difference among patients with infection septic patients for: -Cardiovascular disease -Liver disease -Kidney disease -Hypertension -Diabetes
[22]	Monocyte distribution width	Not applicable (48 h post ICU admission)	Not applicable	Not applicable
[24]	Presepsin Procalcitonin	Not applicable (on admission day)	Not applicable	Not applicable
[25]	Procalcitonin Proadrenomedullin	Within 12 h of ED admission	Not applicable	Not applicable
[26]	Procalcitonin Lactate	Within 1 h of ED admission	Not applicable	Patients comorbidities such as acute heart failure, malignancies, bone marrow transplant history were excluded
[27]	CRP	Within 15 min of ED admission	Not applicable	Not applicable
[28]	CRP	Before operation and daily postoperatively	Not applicable	Not applicable
[29]	CRP	6 h before to 1 h after ICU admission	Not applicable	Not applicable
[30]	Presepsin	At hospital admission	Not applicable	Not applicable
[31]	Soluble FAS Granulysin Interleukin-6 Interleukin-10	For critical cases: upon admission to the ICU and 2, 4, 6, 8, 10 post admission	Not applicable	Not applicable

Abbreviations: ED: emergency department; ICU: intensive care unit

### Biomarkers for early recognition of sepsis in COVID-19

#### Presepsin

Presepsin was measured in 173 hospitalised patients with acute pancreatitis or with infections and at least one sign of the qSOFA. This study included two validation cohorts: 57 admissions to the ED with at least one qSOFA sign, and 115 patients with confirmed cases of COVID-19. In the derivation cohort, blood concentrations above 350 pg/ml had 80.2% sensitivity for sepsis diagnosis (adjusted odds ratio 4.47; *p* < 0.0001). This was 93.3% in the first validation cohort and 78.3% in the second validation cohort [30].

#### Cytokines

A panel of pro- and anti-inflammatory cytokines were measured in a study of 75 COVID-19 patients; 25 were asymptomatic healthy relatives living with the patients; 25 had moderate disease; 27 had severe disease; and 25 had critical disease. Patients with critical illness had higher levels of IL-6, IL-10, and soluble Fas (sFas) than patients with moderate illness (*p* < 0.0001, *p* < 0.0001 and *p* = 0.0031, respectively), as well as contacts (*p* < 0.0001 for the three cytokines). Patients with severe disease also had greater levels of all three cytokines than contacts (*p* = 0.0018, *p* < 0.0001, and *p* = 0.0005, respectively), while patients with moderate disease had higher levels of sFas than the contacts (*p* = 0.0351). Granulysin and IL-10 were higher in patients who were critical (*p* < 0.0001 and *p* = 0.0026, respectively) and severe (*p* = 0.0197 and *p* = 0.0470, respectively) than in contacts, while moderate patients had higher levels of IL-10 than in contacts (*p* = 0.0494) [31] (Table 3).

**Table 3:** Summary of original studies for biomarkers of early sepsis recognition in COVID-19 infection

Reference	Biomarker(s)	Measurements	SIRS, Sepsis and Septic shock definition	Study population	Results
[30]	Presepsin	PATHFAST assay (Mitsubishi, Japan)	Sepsis-3	3 cohorts: derivation cohort: 173 hospitalized patients, validation cohort 1: 57 patients admitted to ED, validation cohort 2: 15 admitted patients with Covid-19 pneumonia	Prediction of severe COVID-19: AUC:0.812, *p* < 0.001 Discriminition of moderate and severe disease: Cut-off value: 12.9 µg/mL, 83% sensitivity and 76% specificity
[31]	sFas, Granulysin, IL-6, IL-10	Bead-based immunoassays (LEGEND-plex, Biolegend)	Sepsis-3	25 asymptomatic household contacts, 25 moderate COVID-19 patients, 27 severe cases, and 25 critical cases. The critical cases were admitted to the ICU.	Higher PCT was strongly associated with death in COVID-19 patients. A PCT cutoff of 0.1 µg/L had high sensitivity (90%) but low specificity (48%) for predicting mortality. sFas: High sensitivity and specificity to differentiate covid-19 survivors with non-survivors. Granulysin: sensitivity 76.9%, specificity 51.9% (optimal cutoff value,12.3 pg/ml) to distinguish survivors from non-survivors. IL-6: A sensitivity of 73.1% and specificity of 78.6% in predicting mortality. IL-10: A sensitivity of 80.8% and specificity of 85.7% in predicting mortality.

Abbreviations: AUC: area under the curve; ED: emergency department; ICU: intensive care unit; IL-6: interleukin-6; IL-10: interleukin-10; PCT: procalcitonin; sFAS: soluble Fas

## Biomarkers to guide antibiotic treatment

Early start of antibiotics is the mainstay of sepsis management [27, 32]. However, prolonged antibiotic use may lead to unfavorable outcomes such as extended hospitalization and increased mortality [32]. The Global Burden of Disease reported that in 2019, antibiotic resistance resulted in 1.3 million of deaths globally [33]. Biomarkers play a critical role in guiding both the initiation and duration of antibiotic therapy [34].

### Biomarkers for early initiation of antibiotic treatment

#### C-reactive protein

Christensen et al. investigated prospectively the contribution of various biomarkers for the early recognition of sepsis in ED. The authors recommended 31 mg/L and 52 pg/mL as thresholds for CRP and IL-6, respectively, regardless of the qSOFA score [27].

#### Soluble urokinase plasminogen receptor

Adami ME et al. investigated whether combining the quick Sequential Organ Failure Assessment (qSOFA) score with the biomarker soluble urokinase plasminogen activator receptor (suPAR) could improve early risk detection and guide antibiotic treatment in ED admissions with suspected infection. The study had two parts. The first part involved the analysis of data from 2,377 patients from the Hellenic Sepsis Study Group (HSSG) registry, classifying them into four groups: 590 patients had no qSOFA signs (Group A), 615 patients had one qSOFA sign and suPAR < 12 ng/mL (Group B), 290 patients had one qSOFA sign and suPAR ≥ 12 ng/mL (Group C), and 882 patients had two or three qSOFA signs (Group D). The mortality was 7.5% (95% Cl: 5–10%) for Group A, 11.5% (95% Cl: 9–14%) for Group B, 30.0% (95% Cl: 25–35%) for Group C and 38.7% (95% Cl: 35–42%) for Group D. Patients in Group C had a significantly increased risk of death (HR: 2.98, 95% CI: 2.11–3.96) compared to group B and had risk of death similar to Group D (HR: 3.99, 95% CI: 3.08–5.16).

The results pointed out that when qSOFA is one and there is uncertainty on the outcome, the use of suPAR may be used for clarification. This guided the second part of their study, namely the SUPERIOR prospective randomized double-blind, controlled trial in two Greek hospitals from November 2018 to December 2020. A single intravenous dose of 2 g meropenem or a placebo was administered to 91 adult ED patients with a suspected infection, one qSOFA sign, and suPAR ≥ 12 ng/mL. The primary endpoint, early clinical worsening (defined as a one-point increase in SOFA score within 24 h) emerged in 40.4% of the placebo group versus 15.9% of the meropenem group (difference: 24.5%, 95% CI 5.9–40.8; OR: 0.14, 95% CI 0.04–0.50, *p* = 0.011). Post hoc analysis showed significant reductions in SOFA scores at 72 and 96 h in the meropenem group [35].

### Biomarkers for response to treatment and early discontinuation of antibiotics

The 2005 prospective observational cohort study by Póvoa et al. was among the first to explore the potential use of biomarkers for monitoring disease progression and treatment response. The authors investigated serial measurements of CRP to indicate improvement or not ventilator-associated pneumonia (VAP). Results suggested that marked decrease of CRP should be considered in decision-making for early stop of antibiotics [36].

#### C-reactive protein

In an observational study by Perrella et al., the role of CRP for the evaluation of efficacy of antibiotics after major abdominal surgery, was investigated. They compared 140 patients with non-emergency major abdominal surgery and no sign of infection with 120 patients with proven microbial infection perioperatively or postoperatively. CRP decreased within the first 48 h, in patients who responded to antibiotics [37].

#### Presepsin

Xiao H et al., evaluated presepsin to guide stop of antibiotics in sepsis in a multicenter prospective cohort. Patients with presepsin ≥350pg/ml were allocated to the presepsin (327 patients) or control (329 patients) groups. In the presepsin group antibiotics stopped when presepsin was < 350pg/ml or decreased by 80% or more compared to the baseline level, for two serial days. Patients of the presepsin group experienced 3.64 less days of antibiotic treatment [32].

#### Procalcitonin

In the seminal SAPS (Stop Antibiotics on Procalcitonin guidance Study) trial, procalcitonin was used to guide early stop of antibiotics when PCT decreased by 80% or more from baseline or when PCT was less than 0.5 ng/ml. In comparison with the standard-of-care (SoC) group, the PCT guidance group experienced shorter antibiotic treatment duration and lower 28-day (*p* = 0.0122) and 1-year (*p* = 0.0158) mortality [38].

In the PROGRESS randomized trial in Greece 256 patients meeting the Sepsis-3 definitions were randomized (1:1) to either the SoC group (*n* = 131), or the PCT guided group (*n* = 125). The PCT rule for early discontinuation of antibiotics was similar to that used in the SAPS study, and antibiotics were discontinued according to PCT criteria by day 5 after initiation. The primary endpoint was the rate of infection-associated adverse events at Day 180 which was defined as death by baseline infection and/or incidence of new infection by multidrug-resistant microorganisms (MDRO) or *Clostridioides difficile*. This composite endpoint was attained in 7.2% of patients allocated to the PCT-guided group compared to 15.3% of the SoC group (hazard ratio 0.45, *p* = 0.045). 28-day mortality was also lower (15.2% in the PCT group versus 28.2% in the SoC group, HR 0.51, *P* = 0.02). PROGRESS provided an association between the attainment of the primary endpoint and gut colonization by MDRO and/or *C.difficile*. The decrease in the incidence of antibiotic-associated adverse events in the PCT-guided group was provided as an explanation for the survival benefit [39].

Fugit et al. assumed that PCT monitoring is neglected often by physicians and supervision of adherence to PCT protocol by the antimicrobial stewardship team (AST) could maximize the positive results. A monocentric before-and-after intervention study was designed and the SAPS algorithm for PCT antibiotic guidance was utilized. 35 ICU septic patients were randomized to the SPAP (standard procalcitonin algorithm period) group and 53 ICU septic patients to the ASPAP (antimicrobial stewardship team-supported procalcitonin algorithm period) group. In 25 out of 57 patients of ASPAP group, antibiotics were withheld in accordance with PCT algorithm; this happened in only 2 out of 25 patients of the SPAP group (*p* < 0.0001) [34].

In another prospective randomized trial from Egypt, investigators used PCT or CRP to guide antibiotic therapy. In 30 out of 60 septic patients, the protocol guided clinicians to initiate or discontinue antibiotic treatment based on PCT less than 0.5 ng/ml or ≥ 80–90% drop from the initial value. Antibiotic treatment was discontinued in 33% septic patients of the PCT group on day 4 and in only 10% of septic patients in the CRP group (*p* = 0.009). More patients from CRP protocol were under antibiotic treatment for over 7 days compared to septic patients from PCT protocol (*p*≤0.0001). 28-day mortality rates were comparable among the two groups (*p* = 0.063) [40].

A summary of original studies for biomarkers which guide antibiotic administration is provided in Table 4.

**Table 4:** Summary of original studies for biomarkers which guide antibiotic administration

Reference	Biomarker	Measurements	SIRS, Sepsis and Septic shock definition	Study population	Results
[27]	CRP IL-6	CRP: Roche Cobas 8000 c702 IL-6: Roche Cobas 8000 e801	Patients with possible sepsis	Admissions to ED were catecorized as: “not likely” group: 154 patients with no signs of infection “definite” group: 102 patients with a microbiologically confirmed infection “probable” group: 135 patients with signs of infection, not microbiologically confirmed	CRP < 31 mg/L and IL-6 < 52 pg/ml and qSOFA < 2: no antibiotics CRP ≥ 31 mg/L and IL-6 ≥ 52 pg/ml and qSOFA < 2: antibiotics CRP ≥ 31 mg/L or IL-6 ≥ 52 pg/ml and qSOFA < 2: examine the use antibiotics CRP < 31 mg/L and IL-6 < 52 pg/ml and qSOFA ≥ 2: re-examine the use of antibiotics
[32]	Presepsin	PATHFAST; Mitsubishi Chemical Medience Corporation, Tokyo, Japan	Sepsis-3 criteria	656 septic patients with presepsin≥350pg/mL. 327/656 compromised the presepsin group and antibiotic treatment was discontinued according to presepsin levels	28-day survival: HR 0.96 (90% CI 0.71–1.31) 90-day survival: HR 1.01 (90% CI 0.76–1.36)
[34]	PCT	N/A	N/A	35 ICU septic patients SPAP group 53 ICU septic patients ASPAP group	Discontinuation of antibiotics per algorithm: SPAP 2/35, ASPAP 25/57, *p* < 0.0001 Total of antibiotic days (median): SPAP 7, ASPAP 5, *p* = 0.02 ICU LOS: *p* = 0.4 Total ICU: *p* = 0.44 30-day mortality: *p* > 0.99 30-day readmission: *p* > 0.99
[35]	suPAR	Flow immunoassay, rapid suPARnostic Quick Triage (ViroGates, Denmark)	qSOFA	91 adult ED patients with suspected infection, one qSOFA sign, and suPAR ≥ 12 ng/mL, who were randomized 1:1 to receive either a single intravenous dose of 2 g meropenem or a placebo.	Early clinical worsening (one-point increase in SOFA score within 24 h), occurred in 40.4% of the placebo group versus 15.9% of the meropenem group. Post hoc analyses showed significant decrease in SOFA scores at 72 & 96 h in the meropenem group.
[37]	CRP	N/A	N/A	140 patients with non-emergency major abdominal surgery and no infection 120 patients with proven microbial infection perioperatively or postoperatively	CRP > 250 mg/L lower rate of effective treatment at 14 days vs. CRP < 250 mg/L *p* < 0.05 1st postoperative day: ↑CRP levels in patients who received Fosfomycin (*p* = 0.001) 2nd and 3rd postoperative day: ↓CRP levels in patients who received Fosfomycin (*p* = 0.0003 and *p* = 0.0001)
[38]	PCT	Kryptor machine [Thermo Fisher Scientific, Waltham, MA, USA] or a suitable Vidas [Marcy-l’Étoile, France] or Roche [Basel, Switzerland] immunoanalyser)	N/A	538 ICU patients: PCT-guided group 457 ICU patients: standard-of-care group	Antibiotic treatment: 5 days PCT group, 7 days standard-of-care group, *p* < 0.00001 PCT group lower 28-day (*p* = 0.0122) and 1-year (*p* = 0.0158) mortality rates
[39]	PCT	VIDAS assay (lower detection limit 0.05 mg/L; bioMérieux)	Sepsis-3 criteria	256 septic patients: 131 patients SOC group 125 patients PCT discontinuation protocol (PCT at day 5 ↓ ≥80% from baseline or < 0.5 µg/L.	The primary outcome of infection-associated adverse events at Day 180 was 7.2% for PCT-guided group vs. 15.3% for SOC group (HR 0.45, *p* = 0.045). 28-day mortality was 15.2% for PCT group vs. 28.2% for SOC group, (HR 0.51, *p* = 0.02).
[40]	PCT CRP	PCT: by enzyme linked immunosorbent assay (ELISA), kit PCT, Sigma-Aldrich, USA CRP: by immunoturbidimetry, kit by the Diagnostic Product Corporation (USA)	Sepsis-3 criteria	60 septic patients: 30 patients: CRP discontinuation protocol (cutoff value < 8.7 mg/L or ↓ ≥50% from admission) 30 patients: PCT discontinuation protocol (cutoff value < 0.5 ng/mL or ↓ ≥80–90% from admission)	Discontinuation of antibiotics on day 4: CRP protocol: 2/30 septic patients PCT protocol: 10/30 septic patients, *p* = 0.009 Antibacterial treatment ≥ 7 days: CRP protocol: 25/30 septic patients PCT protocol: 10/30 septic patients, *p*≤0.0001

Abbreviations: ASPAP: antimicrobial stewardship team-supported procalcitonin algorithm period; CRP: C-reactive protein; ED: emergency department; HR: hazard ratio; ICU: intensive care unit; IL-6: interleukin-6; N/A: not applicable; PCT: procalcitonin; qSOFA: quick sequential organ failure assessment; SPAP: standard procalcitonin algorithm period, suPAR: Soluble Urokinase Plasminogen Activator Receptor

## Biomarkers and selection of fluids

Fluid resuscitation aims to restore vascular volume, improve tissue perfusion, and prevent organ failure [41]. The restoration of intravascular volume, cardiac output, and oxygen supply guide the need for fluid resuscitation [42, 43]. Patients with hypoperfusion and sepsis or septic shock should be administered at least 30 milliliters per kilogram of intravenous (IV) fluids during the first three hours of treatment, according to the Surviving Sepsis Campaign guidelines [44].

Dipeptidyl Peptidase 3 (DPP3) is a zinc-dependent aminopeptidase that cleaves dipeptides from the N-terminus of oligopeptides, including important bioactive molecules such as angiotensin II (Ang II) [45]. Although DPP3 is typically an internal enzyme involved in the regulation of oxidative stress, it can be released into the bloodstream in severe cell death or injury, such as sepsis or shock [46]. Angiotensin II (Ang II) is a key hormone in the renin - angiotensin system (RAS), promoting fluid balance, blood pressure maintenance, and vasoconstriction [47]. Once in the bloodstream, circulating DPP3 (cDPP3) quickly breaks down Ang II, causing hemodynamic instability, myocardial depression, and vasoplegia [48].

In the prospective multinational AdrenOSS-1 study, Blet et al. examined the association between cDPP3 levels and short-term outcomes in 585 individuals with sepsis or septic shock. A cutoff value of 40.4 ng/mL was used to stratify study participants. Patients with cDPP3 > 40.4 ng/mL had higher 28-day mortality (41.4% versus 15.1%), increased incidence of acute renal injury (77.9% versus 56.6%), decreased urine output during the first 24 h (median 600 mL against 1130 mL, *p* < 0.0001), and greater need for organ support. Furthermore, they received a higher volume of fluids in the first 24 h (median 2398 mL versus 1800 mL, *p* = 0.0059). When cDPP3 levels decreased to ≤ 40.4 ng/mL the first 24 h, mortality was lower (HR: 0.18, 95% CI: 0.08–0.41). These findings imply that elevated levels of cDPP3 are associated with early signs of renal and circulatory dysfunction and may identify patients at higher risk of fluid resuscitation [49].

The retrospective study of Yang et al. marked the 72-hour fluid balance as an independent risk factor for mortality in cancer patients with sepsis (*P* < 0.01) with a cut-off value of fluid balance = 75.9 mL/kg, demonstrating 81% sensitivity and 77% specificity [50]. Another observational study examined the relationship between intravenous (IV) fluid volume and endothelial glycocalyx (EG) shedding, in 86 septic patients. The study did not show any significant association between IV fluid volume and glycocalyx biomarkers including Syndecan-1 (Syn-1), Syndecan-4 (Syn-4) and Hyaluronan. There was a strong correlation between Syn-1 and Syn-4 but not with Hyaluronan. The mean total fluid volume at 24 h was 4,038 ml and vasopressors were initiated in 64% of patients during the first 24 h [51].

Saoraya J et al. conducted a post hoc analysis of a randomized control trial of 95 septic patients, investigating the likelihood of Syn-1 to guide fluid administration. Increased Syn-1 at baseline (T0) and six hours post-resuscitation (T6) correlated with increased fluid administration for 72 h. Notably, there was no correlation between Syn-1 levels and fluid levels administered the first six hours. Higher vasopressor needs (*p* < 0.05) were linked to greater Syn-1 levels, suggesting increased hemodynamic instability [52]. This supports that greater fluid resuscitation and vasopressor assistance are necessary because glycocalyx breakdown leads to vascular dysfunction. Although hypervolemia has been known as a trigger for natriuretic peptide-induced glycocalyx shedding [53], the study did not describe any direct correlation between Syn-1 and N-terminal pro b-type natriuretic peptide (NT-proBNP) levels [52]. Despite an increase in NT-proBNP after fluid resuscitation (*p* < 0.001), Syn-1 levels appeared unaffected by this effect [52] suggesting that inflammatory-driven glycocalyx degradation may be a more dominant factor than hypervolemia alone [54].

Table 5 summarizes studies evaluating the relationship between biomarkers and fluid administration.

**Table 5:** Summary of original studies for biomarkers which guide selection of fluids, vasopressors and immunotherapy

Reference	Biomarker	Measurements	Sepsis definition	Study population	Results
[49]	cDDP3	Study sponsor	Sepsis-2	585 sepsis patients in the ICU	cDPP3 levels (cut-off value > 40.4 ng/ml) correlated with more fluids upon ICU admission. Patients with high cDDP3 had lower urite output (*p* < 0.0001)
[50]	BNP	N/A	Sepsis-3	233 cancer patients with sepsis, 190 patients were in the survival group & 43 patients were in the death group (28-day mortality).	2-hour fluid balance is an independent risk factor for mortality in (*P* < 0.01) with a cut-off value of fluid balance = 75.9 mL/kg, & 81% sensitivity and 77% specificity.
[51]	EG	Enzyme-linked immunosorbent assay (ELISA)	Sepsis-2	86 adult patients undergoing haemodynamic resuscitation for suspected septic shock in the emergency department	No significant association between IV fluid volume and glycocalyx biomarkers including Syndecan-1 (Syn-1), Syndecan-4 (Syn-4) and Hyaluronan.
[52]	Syndecan-1	Enzyme-linked immunosorbent assay (ELISA) kit (Abcam, Cambridge, MA, USA)	Sepsis-3	95 adult patients with sepsis-induced hypoperfusion who presented to the ED.	Elevated Syn-1 levels at both (T0) and (T6) correlated with increased fluid administration over 24 and 72 h (*p* < 0.05). higher vasopressor needs (*p* < 0.05) were linked to greater Syn-1 levels
[56]	BNP	Chemiluminescence immunoassay	Sepsis-3	162 Septic shock patients	BNP and cTnI levels were decreased after NE therapy. EF% improved the most in patients who received NE two hours after fluid resuscitation. Decreased survival was linked to higher baseline BNP and cTnI levels
[61]	Lymphocytes	N/A	Sepsis-1	27 patients with septic shock and lymphocytes count≤900/µl: Placebo group: 10 patients 17 patients received CYT107 (8 patients in low frequency and 9 patients in high frequency)	Increased absolute lymphocyte count after 4 weeks of CYT107 treatment group (*p* < 0.001) and 2–4 weeks after treatment discontinuation (*p* < 0.001)
[62]	FCGR2C	N/A	Sepsis-3	53 septic survivors 28 septic non-survivors	different expression between the two groups
[64]	IFN-γ CD8 cell count	IFN-γ: Ella automated immunoassay system (Bio-Techne, USA)	Sepsis-3	107 patients with sepsis 137 patients with trauma 109 surgical procedure 175 healthy volunteers	↓IFN-γ production Day1-2, Day3-4, Day5-7 IFN-γ for adverse ourcome Day 30:
[66]	suPAR	suPARnostic Quick Triage kit (Virogates)	N/A	594 moderate/severe COVID-19 patients with suPAR levels ≥ 6 ng/ml: Placebo group: 189 patients Anakinra group: 405 patients	Anakinra group: reduced risk of a high 28-day WHO-CPS score: OR 0.36, 95%CI 0.26–0.50, *p* < 0.0001 lower death rates by day 28: HR 0.45, 95%CI 0.21–0.98, *p* = 0.045

Abbreviations: BNP: brain natriuretic peptide; cDDP3: circulating dipeptidyl peptidase 3; cTnI: cardiac troponin I; CYT107: recombinant human IL-17; ED: emergency department; EF: ejection fraction; EG: endothelial glycocalyx; HR: hazard ratio; FCGR2C: Fc gamma receptor 2 C gene; ICU: intensive care unit; IFN-γ: interferon gamma; N/A: not applicable; NE: norepinephrine; OR: odds ratio; Syn-1: Syndecan-1; Syn-4: Syndecan-4; WHO-CPS: World Health Organization’s Clinical Progression Scale

## Biomarkers and vasopressors

Vasopressor therapy, which tries to improve organ perfusion pressure by correcting the vascular tone depression, is a basic treatment for septic shock-induced hypotension in addition to fluid resuscitation [55]. A retrospective analysis of 162 septic shock (SS) patients was conducted by Kang et al., examining the best time to administer norepinephrine (NE) and sodium phosphocreatine (SP) to enhance cardiac function and survival. Patients were divided into four groups: NE administration at one hour (NE-1 h), two hours (NE-2 h), and three hours (NE-3 h) after fluid infusion, as well as a group receiving NE at 2 h in combination with sodium phosphocreatine (NE + SP). BNP (brain natriuretic peptide) and cTnI (cardiac troponin) levels were elevated in the blood of patients with septic shock and decreased following NE therapy [56].

In comparison to both the NE-1 h and NE-3 h groups, patients in the NE-2 h group exhibited better outcomes, with considerably lower BNP levels (528.2 ± 30.2 ng/L) and cTnI (0.37 ± 0.06 ng/mL), increased ejection fraction (EF%, 49.98 ± 1.94%), and lower pressure-adjusted heart rate (PAR, 11.32 ± 0.28). The NE-2 h group had 28-day mortality rate of 39.6%, which was significantly lower compared to the NE-1 h (62.5%) and the NE-3 h (60.0%) groups (*p* = 0.002 and *p* = 0.040, respectively) (Table 5).

## Biomarkers and immunotherapy

Sepsis is considered a dysregulated host response to infection, since dysregulations in both the innate and adaptive immune responses are observed. Each patient, influenced by factors such as age, comorbidities, environmental exposures, and microbiome, exhibits a distinct immune profile and may experience either hyperinflammation or immunosuppression [57].

### Corticosteroids

A recent meta-analysis by Smit et al., investigated the efficacy of adjuvant corticosteroids for community-acquired pneumonia. Hospitalized patients received corticosteroids (intravenously or orally) within 12–96 h of hospital admission for 7 days. The 30-day mortality was significantly decreased. CRP emerged as the strongest predictor of treatment efficacy, since patients with baseline CRP above 204 mg/L experienced maximum benefit from corticosteroid treatment [58].

### Nivolumab

Major key point in sepsis is the suppressed T-cell activity caused by the upregulation of programmed cell death protein (PD-1) and its ligand (PD-L1). Subsequently, this results in increased apoptosis and reduced production of IFN-γ. Nivolumab, a human immunoglobulin G4 that blocks the interaction of PD-1 with its ligands (such as PD-L1) has been used for cancer treatment. In 2019, Hotchkiss et al. investigated the potential therapeutic role of nivolumab in 31 ICU patients with sepsis. No indications of safety concerns or symptoms consistent with a cytokine storm were observed [59]. Taking into consideration nivolumab’s high cost (27660$/single dose of 960 mg), van den Haak DAC et al. investigated the optimal dose of nivolumab for sepsis treatment. The authors proposed that future clinical studies should aim 20 mg as a single dose, since it was found to be sufficient for immune function restoration during sepsis [60].

### CYT107

In a prospective, multicenter randomized, double-blind phase IIb study (IRIS-7), 27 patients with septic shock and lymphocytes count ≤900/µl were randomized to treatment with placebo or CYT107 (recombinant human IL-7). Absolute lymphocyte count was restored in the CYT107 group (after 4 weeks of treatment) and this effect remained for at least 2–4 weeks [61].

### Fc gamma receptor 2 C gene

By analyzing data from gene expression omnibus (GEO) database and designing a validation cohort, Liu et al. aimed to identify new immune biomarkers that could predict sepsis outcome. Among the 140 genes analyzed, altered expression of the Fc gamma receptor 2 C gene (*FCGR2C*) was identified. *FCGR2C* may contribute to cytotoxic lymphocyte function, since high levels are associated with decreased cytotoxic lymphocytes [62].

### The role of interferon gamma

Secondary infections are a consequence of sepsis-induced immunoparalysis (SII). By analyzing blood samples collected on the 1st, 4th and 7th day post admission, a notable drop in the ratio of lymphocytes to leukocytes and early activation of Treg cells was observed [63]. The prospective longitudinal observational study REALISM study (REAnimation Low Immune Status Marker) served as the groundwork for a new retrospective data analysis. The primary aim was to investigate how interferon gamma (IFN-γ) and CD8 cell counts are associated with adverse outcomes. The study included ICU patients with sepsis, trauma or surgery. For IFN-γ assessment, the Interferon-Gamma release assay (IGRA) with Staphylococcal enterotoxin B (SEB) as a promoter, was implemented. The results demonstrated that patients with severe injuries had lower levels of IFNγ production and multivariate analysis revealed independent IFNγ association with unfavorable outcomes during the entire study period (Days 1–2, Days 3–4, and Days 5–7 post ICU admission) [64].

Based on previous findings regarding reduced production of IFN-γ prior to and during hospital-acquired pneumonia (HAP), the multicenter, placebo-controlled, randomized trial by Roquilly et al. examined the preventive role of interferon gamma-1b in reducing the incidence of HAP in mechanically ventilated patients. Safety considerations led to premature termination of the trial [65].

### Biomarkers and immunotherapy in COVID-19

The phase-3 double-blind randomized controlled trial SAVE-MORE showed how soluble urokinase plasminogen activator receptor (suPAR) guided early anakinra treatment in COVID-19 patients in need of oxygen. 594 patients with suPAR levels ≥ 6 ng/ml were randomized to placebo (189 patients) or anakinra treatment (405 patients). Patients in the anakinra group presented a reduced risk of 28-day clinical deterioration (based on World Health Organization’s Clinical Progression Scale, WHO-CPS) compared to placebo group (OR 0.36, 95%CI 0.26–0.50, *p* < 0.0001). In addition, the 28-day mortality rate was lower in the anakinra group (HR 0.45, 95%CI 0.21–0.98, *p* = 0.045) [66] (Table 5).

## Clustering techniques

According to recent studies, clustering techniques introduce a novel approach to biomarker research, by revealing unique biological signatures, thus enhancing diagnostic precision and therapy stratification. In a recent effort to extend ARDS phenotyping into sepsis, Sinha et al. applied latent class analysis (LCA) to two large prospective observational sepsis cohorts - VALID (*N* = 1140) and EARLI (*N* = 818) - to evaluate whether two ARDS phenotypes - hypoinflammatory and hyperinflammatory - could also be identified in sepsis. The hyperinflammatory phenotype was associated with elevated pro-inflammatory cytokines, increased vasopressor use, higher incidence of bacteremia, and significantly higher mortality [67].

In addition, the authors conducted a secondary retrospective analysis of two randomized controlled trials - PROWESS-SHOCK (*N* = 1680) and VASST (*N* = 778) - applying a validated clinical classifier model (CCM) to assign patients to the same phenotypes. In PROWESS-SHOCK, they observed a differential treatment response to activated protein C: treatment was associated with reduced mortality in hyperinflammatory patients and increased mortality in hypoinflammatory patients, suggesting phenotype-specific therapeutic effects (*p* = 0.0043). A similar analysis of the VASST trial also identified the two phenotypes, but no significant interaction with vasopressor type was observed (*p* = 0.72) [67].

Also, Seymour et al. applied machine learning, to clinical data from over 60,000 patients with sepsis, in a large-scale retrospective study. They were able to discover four reproducible sepsis phenotypes (Alpha, Beta, Gamma, Delta) with distinct patterns of inflammation, organ failure, and mortality [68]. Likewise, Scicluna et al. found four distinct genomic endotypes (Mars1- Mars4) of sepsis based on immune gene expression, using whole-blood transcriptomic profiling, in their prospective observational cohort study. Furthermore, using data from electronic health records Jiang et al. more recently developed a novel, time-aware soft clustering algorithm for ICU patients, identifying six hybrid sepsis sub-phenotypes based on evolving patterns of dysfunction in the lung, liver and kidneys. They also used logistic regression to develop an early-warning sepsis prediction model [69]. Bhavani et al. identified four unique temperature-based phenotypes derived from temperature trajectories, during the first 72 h of hospital admission [70]. In ED patients, Baghela et al. discovered a set of gene expression signatures indicative of sepsis endotypes [71].

Several studies, like those conducted in all-cause sepsis cases, have used transcriptomics to describe the precise gene expression events dysregulated in patients with severe COVID-19. Baghela et al. performed transcriptomic analysis of 124 individuals with confirmed COVID-19 infections. Between severity categories, mechanisms linked to COVID-19 severity were found (ranging from moderate illness to mechanical ventilation and death), and existing sepsis signatures were evaluated for dysregulation. Gene expression patterns that reflect pathophysiological events – including mortality, organ dysfunction, and cellular reprogramming – were significantly enriched and predictive of severity and mortality in individuals infected with COVID-19 [72].

## Conclusions

A biomarker should ideally capture the complex interplay of several systems, including metabolic stress, endothelial dysfunction, immunological dysregulation, and inflammation [15]. High sensitivity and specificity, correlation with disease severity, predictive information, reproducibility, change in response to clinical progression, ease of measurement, and cost-effectiveness are all important features of a sepsis biomarker [73]. These elements are especially important when biomarkers are meant to be measured repeatedly to monitor patient’s clinical status [74]. Therefore, for biomarkers to be successfully implemented in clinical practice, a comprehensive strategy that integrates both biological value and operational feasibility is essential [75].

The purpose of this review was to investigate the significance of biomarkers in sepsis diagnosis and treatment options. The review explored how biomarkers can optimize immunomodulatory therapies, direct vasopressor initiation, guide antibiotic stewardship, and aid in fluid resuscitation decisions, ultimately improving patient care.

Despite these advancements, the implementation of biomarkers into clinical practice remains a challenge due to issues related to specificity, cost, and the need to establish cut-off values.

More research about biomarkers is essential in refining sepsis management and optimizing patient outcomes. One future question is how the rapidly advancing technology, such as smartphone-based applications and point-of-care devices, could contribute to the development and implementation of biomarker-driven sepsis management strategies. These innovations have the potential to enhance real-time monitoring, facilitate early diagnosis, and offer personalized treatment (Fig. 3).

Trials that will be completed soon and will provide further insight in sepsis management, are presented in Table 6.

**Table 6:** Pending trials for biomarkers and sepsis management. (source: ClinicalTrials.gov)

Trial Name	ClinicalTrials.gov ID	Sponsor
Personalized Immunotherapy in Sepsis (ImmunoSep)	NCT04990232	Hellenic Institute for the Study of Sepsis
Emapalumab Treatment For Anticipated Clinical Benefit In Sepsis Driven By The Interferon-Gamma Endotype (The EMBRACE Trial)	NCT06694701	Hellenic Institute for the Study of Sepsis
Clarithromycin Treatment to Prevent Sepsis Progression in CAP (REACT)	NCT06294600	Hellenic Institute for the Study of Sepsis
Assessing the Procalcitonin-guidance and Molecular-guided Diagnosis for Therapy of Severe Infections (the MODIFY Trial)	NCT05909683	Hellenic Institute for the Study of Sepsis
Efficacy and Safety of a Protocol Using C-reactive Protein to Guide Antibiotic Therapy	NCT05841875	Federal University of Minas Gerais
BIomarkers to Predict the Outcomes of Sepsis (BIPROS)	NCT05842980	Qilu Hospital of Shandong University
Personalized Swiss Sepsis Study (PSSS_digital)	NCT04130789	University Hospital, Basel, Switzerland
Rapid Recognition of Corticosteroid Resistant or Sensitive Sepsis (RECORDS)	NCT04280497	Assistance Publique - Hôpitaux de Paris
ObsErvational Study of the Practical Cinical UTility of the NuQ.^®^ H3.1 Nucleosome Levels in Adult Patients With Sepsis to Facilitate Early Diagnosis and Prognostication. (EPICETUS)	NCT05922371	Guy’s and St Thomas’ NHS Foundation Trust
Towards Novel BIOmarkers to Diagnose SEPsis on the Emergency Room (BIOSEP)	NCT06178822	Amsterdam University Medical Centers (UMC), Location Academic Medical Center (AMC)

## Electronic supplementary material

Below is the link to the electronic supplementary material.

Supplementary Material 1

## Data Availability

Not applicable.

## Abbreviations

Ang II: Angiotensin II
ARDS: Acute respiratory distress syndrome
ASPAP: Antimicrobial stewardship team-supported procalcitonin algorithm period
AST: Antimicrobial stewardship team
AUC: Area under curve
BNP: Brain natriuretic peptide
BSI: Bloodstream infection
CBC: Complete blood count
CCM: Clinical classifier model
CD8: Cluster of differentiation 8
CD14: Cluster of differentiation 14
cDPP3: circulating Dipeptidyl peptidase 3
CI: Confidence interval
CRP: C-reactive protein
cTnl: cardiac troponin
CYT107: recombinant human IL-7
ED: Emergency department
EF%: Ejection fraction
EG: Endothelial glycocalyx
FCGR2C: Fc gamma receptor 2 C gene
GEO: Gene expression omnibus
HBP: Heparin-binding protein
HDL-c: High-density lipoprotein cholesterol
HR: Hazard ratio
ICU: Intensive care unit
IFN-γ: Interferon gamma
IGRA: Interferon-Gamma release assay
IL-6: Interleukin-6
IL-7: Interleukin-7
IL-10: Interleukin-10
IQR: Interquartile range
IV: Intravenous
LBI: Localized bacterial infection
LCA: Latent class analysis
MDW: Monocyte distribution width
MxA: Myxovirus resistance protein A
NE: Norepinephrine
NEWS: National early warning score
NPV: Negative predictive value
NT-proBNP: N-terminal pro b-type natriuretic peptide
OR: Odds ration
qSOFA: quick sequential organ failure assessment
PAR: Pressure-adjusted heart rate
PCT: Procalcitonin
PD-1: Programmed cell death protein
PD-L1: Programmed cell death ligand 1
PPV: Positive predictive value
proADM: proadrenomedullin
RAS: Renin - angiotensin system
REALISM: REAnimation Low Immune Status Marker
SAPS: Stop Antibiotics on Procalcitonin guidance Study
SEB: Staphylococcal enterotoxin B
sFas: soluble Fas
SIRS: Systemic inflammatory response syndrome
SOC: Standard of care
SP: Sodium phosphocreatine
SPAP: Standard procalcitonin algorithm period
SS: Septic shock
suPAR: soluble urokinase plasminogen activator receptor
Syn-1: Syndecan-1
Syn-4: Syndecan-4
WHO-CPS: World Health Organization’s Clinical Progression Scale
