# Supplementary material for: Biomarkers to guide sepsis management
Source: Ann Intensive Care. 2025 Jul 21;15:103. doi: 10.1186/s13613-025-01524-1 (PMC12277237; doi:10.1186/s13613-025-01524-1)
Supplement: Supplementary file 1 — Supplementary Material 1 [file 13613_2025_1524_MOESM1_ESM.docx]

**Supplementary file**

**Retrieval process:**

**For biomarkers of early recognition**, the search included the terms: (((bacterial sepsis) OR (sepsis[MeSH Terms])) AND (early recognition OR early detection OR early diagnosis OR prompt recognition OR prompt detection OR prompt diagnosis[MeSH Terms])) AND (biomarkers[MeSH Terms]) ((biomarkers[MeSH Terms]) for bacterial sepsis; and (COVID-19 sepsis[MeSH Terms])) AND (early recognition OR early detection OR early diagnosis OR prompt recognition OR prompt detection OR prompt diagnosis[MeSH Terms]) for COVID-19 sepsis. The keywords, for the biomarkers which assist **in the decision regarding antibiotics** (early administration, selection and treatment) in bacterial sepsis or general treatment in COVID-19, were **((bacterial sepsis) OR (sepsis[MeSH Terms])) AND (biomarkers[MeSH Terms])) AND (antibiotics[MeSH** Terms]) and ((biomarkers[MeSH Terms]) AND (COVID-19 sepsis[MeSH Terms])) AND (treatment[MeSH Terms]), respectively. The subject terms **for vasopressors administration** were ((((bacterial sepsis)) OR (sepsis[MeSH Terms])) AND (biomarkers[MeSH Terms])) AND (vasopressors[MeSH Terms]) and ((biomarkers[MeSH Terms]) AND (COVID-19 sepsis[MeSH Terms])) AND (vasopressors[MeSH Terms]) for COVID-19 sepsis. For the **administration of fluids**, the following keywords were used: ((((bacterial sepsis) OR (sepsis[MeSH Terms])) AND (biomarkers[MeSH Terms])) ) AND (administration of fluids OR selection of fluids) for bacterial sepsis and ((biomarkers[MeSH Terms]) AND (covid-19[MeSH Terms])) AND (administration of fluids OR selection of fluids)) for COVID-19 sepsis. Biomarkers which **guide immunotherapy** were searched as following: (((bacterial sepsis) OR (sepsis [MeSH Terms])) AND (biomarkers[MeSH Terms])) AND (immunotherapy[MeSH Terms]) and ((biomarkers[MeSH Terms]) AND (COVID-19 sepsis[MeSH Terms])) AND (immunotherapy[MeSH Terms]). Two authors (VB and EAR) independently read all retrieval abstracts.

**Supplementary Table Combined Eligibility Criteria for Literature Retrieval**

| **Criterion** | **Description** |
| --- | --- |
| Timeframe | January 2020 – November 14, 2024; one relevant 2025 meta-analysis and landmark studies pre 2020 also included. |
| Language | English |
| Population | Adults (≥18 years old) with suspected or confirmed viral or bacterial sepsis. |
| Study types | Original research articles; observational studies (prospective / retrospective), RCTs and meta-analysis. |
| Sample size | ≥ 30 patients |
| Clinical focus | Sepsis management; early recognition, antibiotic guidance, vasopressor initiation, fluid administration, immunotherapy. |
| Biomarker role | Predictive, diagnostic, prognostic, or therapeutic – decision guiding. |
| Setting | ED, ICU, hospital ward |
| Metrics Required | Clinical performance data (e.g. AUC, sensitivity/specificity, hazard ratios, cut-off values). |
| Exclusions | Pediatric studies, in vitro or animal – only studies, case reports, reviews, editorials, letters, consensus statements, outpatient settings, non-English publications. |

Abbreviations: RCTs: randomized control trials; ED: emergency department; ICU: intensive care unit, e.g.: for example, AUC: area under the curve
